# Potential drug incompatibilities in the neonatal intensive care unit: a network analysis approach

**DOI:** 10.1186/s40360-018-0265-7

**Published:** 2018-12-06

**Authors:** Ramon Weyler Leopoldino, Haline Tereza Costa, Tatiana Xavier Costa, Rand Randall Martins, António Gouveia Oliveira

**Affiliations:** 1 Department of Pharmacy, Centro de Ciências da Saúde, Universidade Federal do Rio Grande do Norte, Av. General Gustavo Cordeiro de Farias, s/n. Petrópolis, Natal, RN 59012-570 Brazil; 2 Maternity School Januário Cicco, Centro de Ciências da Saúde, Universidade Federal do Rio Grande do Norte, Av. Nilo Peçanha, 259. Petrópolis, Natal, RN 59012-310 Brazil

**Keywords:** Infant, newborn, Critical care, Drug incompatibility, Patient safety, Network analysis

## Abstract

**Background:**

There is little information on the frequency of drug incompatibilities in neonatal intensive care units (NICU) and the agents most commonly involved in them. The objective of the study was to characterize potential Drug Incompatibilities (DI) in the NICU by frequency, type and combination of drugs.

**Methods:**

Between August 2015 and December 2016, all neonates admitted for more than 24 h and who received any drug treatment were included in this cohort study conducted in the NICU of a teaching maternity hospital in Brazil. Patient data were collected from patient records and prescription orders, and the compatibilities of all drug pairs were classified using the Trissel’s™ 2 IV Compatibility tool. Network analysis was performed in order to visualize the drug pairs commonly involved in potential DI.

**Results:**

The study population consisted of 281 neonates with a median NICU length of stay of 11 days (range 2–184) and received 1343 intravenous medications. A total of 1114 potential DI were identified, 469 (42.1%) were restricted compatibilities, 348 (31.2%) unknown compatibilities and 297 (26.7%) documented incompatibilities. The incidence of documented incompatibilities in the NICU was 25.0% patient-days (95% confidence interval (CI) 19.4–30.7% patient-days). Incompatible potential DI affected 46.3% (95%CI 40.3–52.3%) of the neonates. Ampicillin (408 of 1114 pairs), gentamicin (216 of 1114 pairs) and aminophylline (197 of 1114 pairs) were the main medicines involved in potential DI.

**Conclusion:**

Potential DI are extremely common in NICU, with half of the population susceptible to simultaneous administration of incompatible medications. More research is needed to understand the actual drug incompatibilities and their clinical outcomes.

## Background

Drug Incompatibility (DI) is a reaction between an intravenous drug and the diluent, container or other intravenous drug, causing visible changes or degradation of more than 10% of the drug [1, 2]. Incompatibilities are classified as physical or chemical [1]. Usually readily visible, physical incompatibility reactions are rapid, reversible and can cause precipitate formation, gas release, or changes in viscosity and color [1, 3]. In contrast, drug degradation through chemical incompatibility is predominantly slow, irreversible and not visible as hydrolysis, oxidation or covalent reaction [1, 3].

DI can compromise drug effectiveness and/or patient safety [1]. For example, Foinard et al. [4] observed that concurrent administration of furosemide (10 mg/ml) and midazolam (1 mg/ml) at the same infusion rate (2 mg/h) resulted in 14% furosemide precipitation. The precipitate formed can reach the bloodstream and compromise tissue perfusion and function of vital organs [5]. Moreover, there fatal cardiopulmonary complications due to precipitation between ceftriaxone and calcium electrolyte solutions have been documented in neonates [6].

In the hospital setting, reactions between medications (actual drug incompatibility) are poorly observed, but circumstances that favor contact between incompatible medications or between medication with poorly documented compatibility (potential drug incompatibility) are common [7–9]. A Canadian study involving 434 intensive care adults showed a potential DI prevalence of 8.5% [10]. Another Canadian study found a potential DI prevalence of 52% in more than 16,000 pediatric patients [11].

It is believed that neonates in the Neonatal Intensive Care Unit (NICU) have greater exposure to DI, as compared to adult patients. Neonates typically have a single access route for intravenous administration, which increases the chance of drug mixing. Additionally, the volume of diluents is smaller and the infusion rate is slower than in adults, which might lead to high concentrations and prolonged contact time between incompatible drugs [12]. However, information on the frequency and type of DI within the NICU is lacking.

As a contribution to closing this knowledge gap, the objective of this study was to characterize potential DI in NICU according to their frequency, type and to identify the most common inappropriate combination of drugs through network analysis.

## Methods

This prospective cohort study was conducted during a 17-month period, from August 2015 to December 2016, in the 20-bed NICU of a teaching maternity hospital specialized in high-risk pregnancy, in Brazil. All neonates admitted to the NICU for more than 24 h and who received drug treatment were included in the study.

The data were collected from the clinical patient records and the prescription orders. Clinical and demographic data (sex, gestational age, birth weight and length of stay) were recorded from every newborn included in the study, as well as every intravenous medication prescribed throughout NICU stay. Electrolyte and parenteral nutrition solutions, diagnostic agents and vitamin and mineral supplements were not considered as medicines.

A daily evaluation of the compatibility of all prescribed drugs of each neonate was done with the Trissel’s™ 2 IV Compatibility tool available in the Micromedex® database (Truven Health Analytics, Michigan, USA). A potential DI was defined as the prescription of two intravenous medicines that were not documented to be compatible. Potential DI were classified according to the quality of the compatibility studies performed: incompatible (well-documented drug interaction), restricted compatibility by concentration and diluent (discordant drug compatibility data), and unknown (no compatibility data). The pairs of incompatible medicines were classified according to the physicochemical mechanism of the reactions as precipitation, turbidity, decomposition and color change. All identified potential DI were reported to the NICU healthcare team for appropriate action.

### Statistical analysis

The data are described as mean ± standard deviation, absolute and relative frequencies, and median and range as appropriate in each case. Potential DI prevalence was defined as the proportion of patients with at least one potential DI during the NICU stay and presented as point estimates and exact 95% confidence intervals (CI). Potential DI incidence is presented as incidence density and defined as number of potential DI as % patient-days with Poisson 95% CI. The risk of potential DI associated with the medicines most commonly involved in potential DI was estimated with logistic regression and presented as odds-ratios (OR) with 95% confidence intervals (CI). The analysis was performed with Stata® 13 (Stata Corporation, College Station, TX, USA). A network analysis was carried out using Gephi 0.9.1 [13] to visualize the medicines and the pairs commonly involved in potential DI. For this analysis, the ForceAtlas2 algorithm [14] was applied.

## Results

During the study period, 430 neonates were admitted to the NICU and 104 of those were excluded because of NICU stay < 24 h (95 patients) or because they had no drugs prescribed (9 patients). From the 326 neonates included in the study, 45 (13.8%) were excluded from the analysis because of missing relevant data items. Therefore, the analysis set included 281 patients that were observed for a total of 5495 days, consisting of 57.3% males, with mean gestational age of 32.6 ± 3.9 weeks and mean birth weight of 1956.5 ± 910.9 g. The median NICU length of stay was 11 days (range 2–184 days). A total of 1343 intravenous medications were prescribed, representing a mean of 4.8 ± 3.7 medications per patient. The most prescribed intravenous medicines, administered to 259 (92.2%) neonates, were gentamicin, ampicillin, aminophylline, fentanyl, cefepime and amikacin. The mortality rate in the NICU was 7.1% (20 deaths). Demographic and clinical characteristics of the study population are summarized in Table 1. Two hundred fifty-nine neonates.

**Table 1:** Characteristics of the study population (*n* = 281)

Demographic and clinical characteristics	Value	
Male sex (*n*, %)	161	57.3
Gestational age in weeks (m, sd)	32.6	3.9
Birth weight in grams (m, sd)	1956.5	910.9
Length of stay in days (median, range)	11	2–184
Number of intravenous drugs (m, sd)	4.8	3.7
Intravenous medicines (*n*, %)		
Gentamicin	226	16.8
Ampicillin	158	11.8
Aminophylline	149	11.1
Fentanyl	114	8.5
Cefepime	70	5.2
Amikacin	69	5.1
Other	557	41.5
Death (*n*, %)	20	7.1

*m* mean, *sd* standard deviation, *CI* confidence interval

A total of 1114 potential DI were identified, representing an average of 19.6 ± 36.4 potential DI per patient. Of these, 469 (42.1%) were restricted compatibility, 348 (31.2%) were unknown compatibility and 297 (26.7%) documented incompatibilities. Two hundred and ten (74.1, 95% CI 69.2–79.7%) neonates had at least one potential DI. The incidence of potential DI was 99.9% patient-days (95% CI 80.9–118.9% patient-days). The prevalence and incidence of neonates with incompatible potential DI was 46.3% (95%CI 40.3–52.3%) and 25.0% patient-days (95%CI 19.4–30.7% patient-days), respectively. Table 2 has more information about the frequency and characteristics of potential DI.

**Table 2:** Frequency and characterization of potential Drug Incompatibilities (DI) in Neonatal Intensive Care

Potential DI	Combinations *n* (%)	Prevalence *n* (%; 95%CI)	Incidence % patient-days (95%CI)
Restricted^a^	469 (42.1)	195 (69.4; 63.6–74.7)	41.7 (35.1–48.4)
Unknown^b^	348 (31.2)	84 (29.9; 24.6–35.6)	33.1 (22.7–43.6)
Incompatible^c^	297 (26.7)	130 (46.3; 40.3–52.3)	25.0 (19.4–30.7)
Total	1114 (100.0)	210 (74.7; 69.2–79.7)	99.9 (80.9–118.9)

*CI* confidence interval

^a^Drug compatibility with restriction by concentration and diluent

^b^Drug compatibility without compatibility data

^c^Drug incompatibilities well documented in the literature

The most frequent pairs of medicines with potential DI are shown in Table 3. The most frequent pairs were Ampicillin – Gentamicin (158, 14.12%), Aminophylline – Ampicillin (77, 6.91%) and Ampicillin – Fentanyl, (61, 5.48%). The median (range) exposure time to each drug pair was 6 days (1–13 days), 5 days (1–11 days) and 5 days (1–11 days), respectively.

**Table 3:** Frequency, days of prescriptions and interaction characteristics of medicine pairs with potential Drug Incompatibility

Medicine pairs	Number	Percent	Days of prescriptions		Interaction characteristics
			Median	Range	
Ampicillin – Gentamicin	158	14.2	6	1–13	A
Aminophylline – Ampicillin	77	6.91	5	1–11	E
Ampicillin – Fentanyl	61	5.48	5	1–11	A
Cefepime – Fentanyl	35	3.14	5	1–11	B
Aminophylline – Cefepime	32	2.87	5	1–10	B
Aminophylline – Dobutamine	28	2.51	3	1–18	D
Ampicillin – Dobutamine	26	2.33	3	1–10	C
Furosemide – Gentamicin	26	2.33	2	1–10	A
Fentanyl – Meropenem	25	2.24	9	2–18	B
Dobutamine – Furosemide	23	2.06	4	1–48	A
Total	1114	100.00	8	1–48	–

A: Restrictions on concentration and infusion fluid, B: There is no data, C: Appearance of precipitation, D: Appearance of turbidity, particle formation and/or color, E: Chemical decomposition

**Fig. 1:**
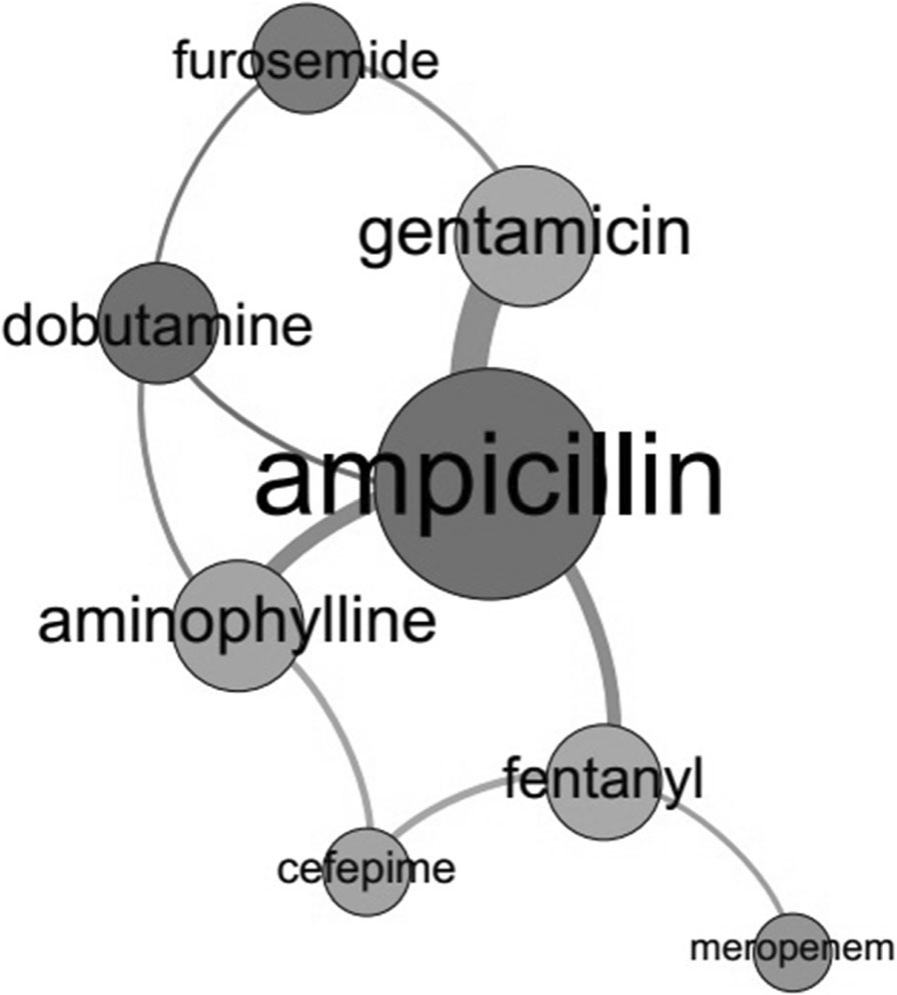
Main medicines involved in potential Drug Incompatibility (DI). The figure shows a graph in which the nodes are medicines and the ties their combinations. The major node and tie represent, respectively, the medicine and combination most often involved in potential DI. In total, medicines were involved in 1114 potential DI. Of these, 408 were related to ampicillin, 216 were related to gentamicin, 197 were related to aminophylline, 174 were related to dobutamine, 162 were related to fentanyl, 150 were related to furosemide, 103 were related to cefepime and 83 were related to meropenem

Figure 1 shows the network analysis of the main medicines involved in potential DI. This figure contains eight nodes (representing the main medicines involved) and ten ties (representing drug combinations). Ampicillin (408 of 1114 pairs), gentamicin (216 of 1114 pairs) and aminophylline (197 of 1114 pairs) were the medicines most involved in potential DI. The estimates of the risk of occurrence of potential DI for the medicines most often implicated were the following: ampicillin (OR 30.9, 95% CI 14.3–67.7), gentamicin (OR 3.3, 95% CI 2.6–4.6), aminophylline (OR 5.0, 95% CI 4.0–7.2), dobutamine (OR 1.3, 95% CI 1.1–1.7), furosemide (OR 74.5, 95% CI 34.4–161.0) and fentanyl (OR 4.9, 95% CI 3.6–6.6).

## Discussion

Our main findings indicate that about 80% of newborns are exposed to potential DI during their stay in the NICU, with an expectation of one potential DI per patient each day. We also found that half of the neonates had at least one potential DI well documented in the literature. Other important results were restricted compatibility and that Ampicillin–Gentamicin was the most common drug pair and also the most common potential DI. The medicines most involved in potential DI were ampicillin, gentamicin and aminophylline.

Very few studies have looked into the characteristics of potential DI in pediatrics. As far as we know, only five papers have studied potential DI in hospitalized children, as the majority of potential DI papers in pediatrics are population-based studies. A retrospective study evaluated more than 16 thousand children between 2006 and 2015 [11], and the other works were cross-sectional surveys conducted in less than 1 year [15, 16]. All the authors identified potential DI through the analysis of the medication orders, only Gikic and collaborators [7] evaluate in loco the occurrence of incompatibilities at the moment of infusion. Works of this nature need adequate sources for the characterization of potential DI. For example, *Trissel’s Handbook on injectable drugs* is the main reference for several authors [7, 9, 11, 15, 16].

Evaluating prospectively close to 300 newborns, we observed that nearly half of these patients had prescriptions with potential DI well described in the literature. Gaetani et al. [11], with a retrospective study in a large pediatric population not including neonates, observed an equivalent prevalence.

The most observed potential DI in our study was compatibility with restriction by concentration and diluent. In contrast, several authors showed unknown compatibility as the main potential DI [7, 11, 15, 16], including Kalikstad et al. [9] who, when analyzing the compatibility of medicines in NICUs, found 60% of drug combinations with unknown compatibility. However, the authors limited themselves to analyzing potential DI related to the most frequent medicines, while we have described the potential combinations of all drugs.

The restricted compatibility observed in most drug combinations in the NICU shows that many compatibility studies have conflicting results and are not very clear, therefore questionable as to quality. These studies are old and inadequate for clinical practice, present different methods and technique, and disregard important parameters such as brand, diluent and concentration of medicines [8]. In addition, the off-label use of medicines in neonatology and the profusion of new products on the market contribute to discordant information and data scarcity [2, 9].

Through network analysis, we were able to map the most relevant medicines to potential DI in the NICU. In this regard, ampicillin clearly plays a central role in the occurrence of potential DI, especially considering its prescription together with gentamicin, dobutamine and aminophylline. Although ampicillin is one of the most commonly used NICU drugs [17], the main problems related to its preparation and administration are pH-sensitive solubility and strong hydrolysis in the presence of dextrose, which requires a lot of attention from the healthcare team [18].

There are three strategies to avoid the simultaneous administration of potentially incompatible drugs: 1) administration of drugs in different venous accesses or at different times, 2) rinsing of the infusion system with a neutral IV solution prior to the application of another drug, or 3) use of a multilumen device [1, 19]. However, the large number of medicines used in intensive therapy can make it almost impossible to administer a single medicine, in particular medicines administered through continuous infusion. On the other hand, additional vascular accesses increase the risk of infections and thromboembolic complications, while not administering one of the drugs compromises treatment effectiveness; therefore, in many situations, the occurrence of drug incompatibility is inevitable [8].

This study has some limitations. Our results derive only from the analysis of prescriptions, and the administration process was not directly observed in order to detect mixture of drugs and the occurrence of incompatibilities. The correspondence of actual to potential drug incompatibilities depends on local practices, on the availability of institutional treatment protocols and on the general organization of the service, for example, whether the administration of medicines is supervised by a clinical pharmacist. In addition, the data were collected in a single institution, which may restrict the generalization of our findings. However, as far as we know, this is the first prospective cohort to investigate and describe drug incompatibility in NICUs. The study design and the use of the Trissel’s ™ 2 IV Compatibility tool, gold standard in compatibility analysis [20], strengthen our results. In addition, we were able to demonstrate the applicability of the network analysis in the visualization of the drugs and pairs most involved in potential DI. Further research should be performed by direct observation of drugs administered in large populations as well as related clinical outcomes.

## Conclusion

In this prospective cohort, we emphasize that potential DI are extremely common in NICU, and half of the newborns are susceptible to simultaneous administration of incompatible drugs. In addition, restricted compatibility is the main potential DI, and Ampicillin–Gentamicin is the most frequent pair. Particular attention should be given to ampicillin, gentamicin and aminophylline, as these drugs were the most commonly involved in potential DI.

## Abbreviations

CI: Confidence Interval
DI: Drug incompatibility
NICU: Neonatal Intensive Care Unit
OR: Odds Ratio
